# Penetrating orbital injury by a nine-centimetre lead sinker

**DOI:** 10.1136/bcr-2025-265477

**Published:** 2025-07-08

**Authors:** Chaoyu Lei, Yinwei Li, Huifang Zhou, Sisi Zhong

**Affiliations:** 1 State Key Laboratory of Eye Health, Department of Ophthalmology, Shanghai Ninth People's Hospital, Shanghai Jiao Tong University School of Medicine, Shanghai, China

**Keywords:** Ophthalmology, Orthopaedic and trauma surgery

## Abstract

A male in his early 40s presented with a penetrating orbital injury after a nine-centimetre lead sinker was propelled into his right orbit while fishing. He reported pain, ptosis and visual acuity reduced to light perception. X-rays, preferred over CT due to metallic artefacts, revealed the sinker in the inferior orbital fissure with an intact eyeball. Initial surgical extraction attempts triggered a severe vagal response, necessitating endoscopic navigation for safe removal. Postoperatively, despite an intact globe, he developed vitreous haemorrhage and retinal detachment, requiring vitrectomy and silicone oil injection. Three months later, his visual acuity improved to 20/200, with normal blood lead levels. This case emphasises selecting imaging based on foreign body material, avoiding blind extraction using advanced tools if needed, monitoring for intraocular complications and assessing systemic toxicity risks.

## Background

 Penetrating orbital injuries are rare yet pose significant challenges due to the orbit’s complex anatomy and its closeness to vital structures like the brain and optic nerve. While fishing-related accidents typically involve smaller objects such as hooks, a nine-centimetre lead sinker piercing the orbit is an exceptionally uncommon and severe case, heightening risks like vision loss, infection or systemic toxicity. This report emphasises the critical need for a meticulous management strategy—including specialised imaging, careful surgical techniques and thorough postoperative follow-up—to effectively address such high-risk, unusual orbital traumas and optimise patient outcomes.

## Case presentation

**Figure 1:**
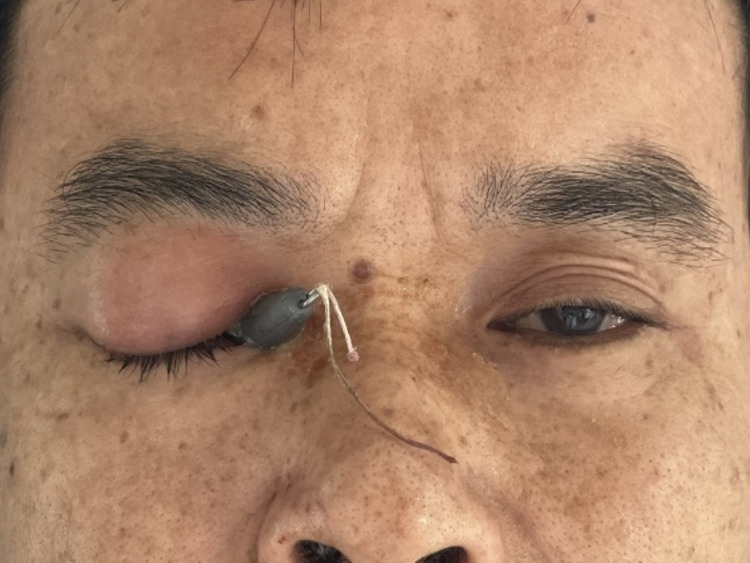
Clinical photograph of the patient’s right eye showing a lead sinker embedded in the orbit through the conjunctiva near the lacrimal mound, with significant swelling and ptosis.

**Figure 2:**
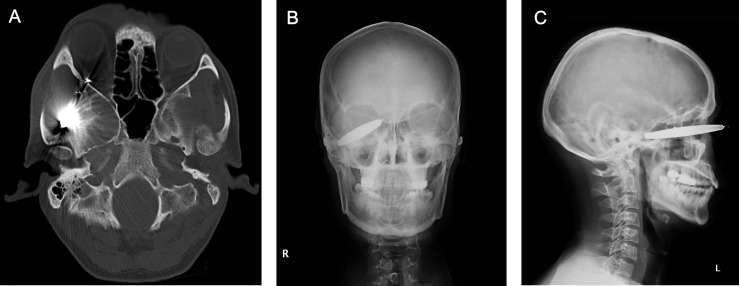
Orbital imaging: (**A**) axial CT scan, (**B**) anteroposterior X-ray and (**C**) lateral X-ray, all showing penetration of the foreign body through the orbital cavity into the inferior orbital fissure, with the globe itself remaining intact.

A male in his early 40s presented to the emergency department with a 15-hour history of right eye pain and difficulty in opening the eye. 15 hours prior to presentation, he accidentally had a lead sinker propelled directly into his own right orbit while fishing without wearing any eye protection device. Physical examination revealed a lead bar inserted into the orbit through the conjunctiva near the lacrimal mound. His visual acuity was reduced to light perception (LP), with pupil distortion, ptosis and restricted ocular motility (figure 1). Intraocular pressure was within normal limits, measured using an iCare rebound tonometer, which helped rule out globe rupture. Due to artefacts from metallic foreign bodies on CT scans (figure 2A), lateral skull X-rays were used (figure 2B,C), clearly demonstrating penetration through the orbit into the inferior orbital fissure, with an intact eyeball. No optic canal fracture was suspected, as orbital CT showed an intact canal and the patient exhibited no clinical signs of optic nerve injury. Fundus examination was not performed preoperatively because the lead sinker obstructed the visual axis, and orbital ultrasound was similarly precluded by complex echoes from post-traumatic haemorrhage and oedema, resulting in poor spatial resolution.^1 2^ Based on the history, clinical findings and X-rays, a diagnosis of intraorbital foreign body was made.

## Treatment

**Figure 3:**
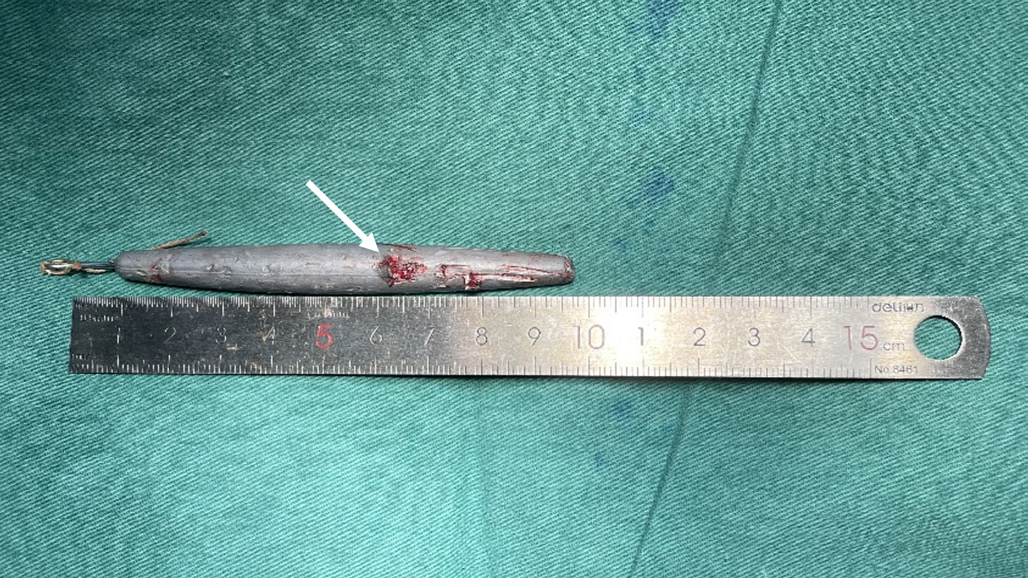
Intraoperative photograph of the removed foreign body, a nine-centimetre lead sinker, following successful surgical extraction using an endoscopic navigation system via a conjunctival approach.

Emergency surgery was performed under general anaesthesia. Initial attempts to directly extract the foreign body after minimal exposure were unsuccessful and led to severe vagal response, with the patient’s heart rate dropping to 20 beats per minute. Extraction was immediately halted, and atropine was administered to stabilise the patient. Given the complexity of the impaction, an endoscopic navigation system was employed to facilitate precise localisation. A transconjunctival approach was used to expose the orbital floor and the infraorbital fissure until the distal end of the foreign body was identified. No orbital wall fractures were observed, but the medial rectus muscle fibres demonstrated mild involvement. The nine-centimetre lead sinker was carefully mobilised and removed in a controlled manner (figure 3). Post-extraction examination revealed a barb-like protrusion on the sinker, likely formed on impact with the orbital bone, which explained the resistance encountered during initial extraction attempts.

## Outcome and follow-up

**Figure 4:**
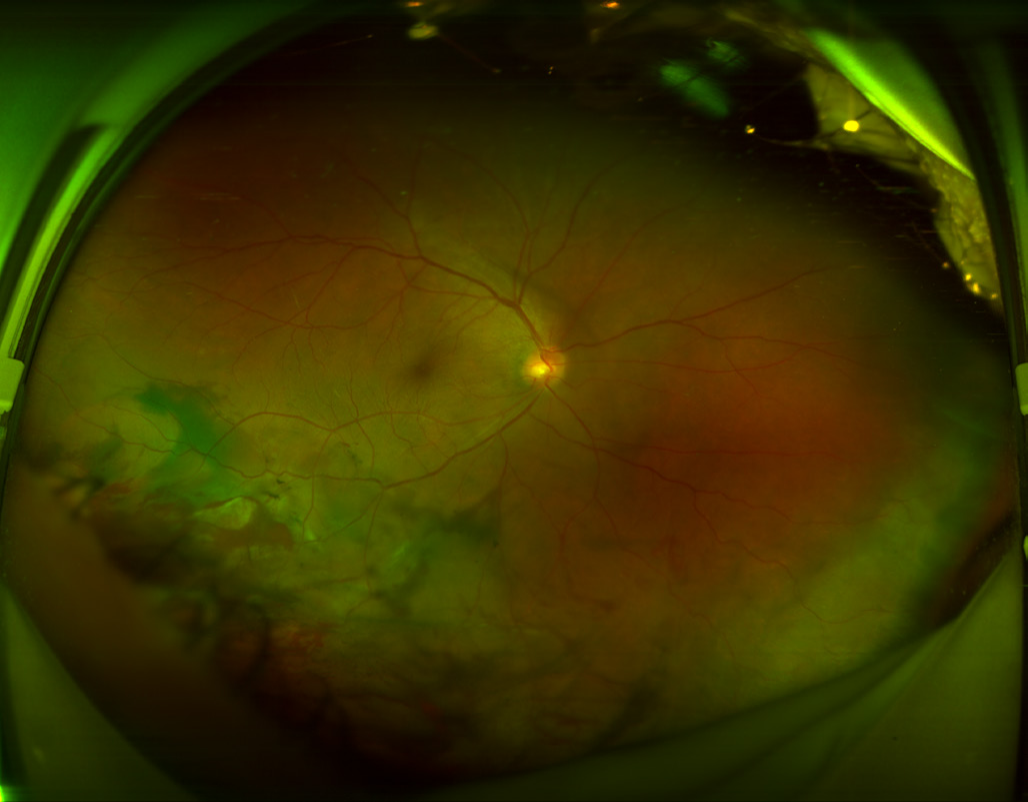
Ultra-widefield fundus photography revealing vitreous haemorrhage and retinal detachment.

**Figure 5:**
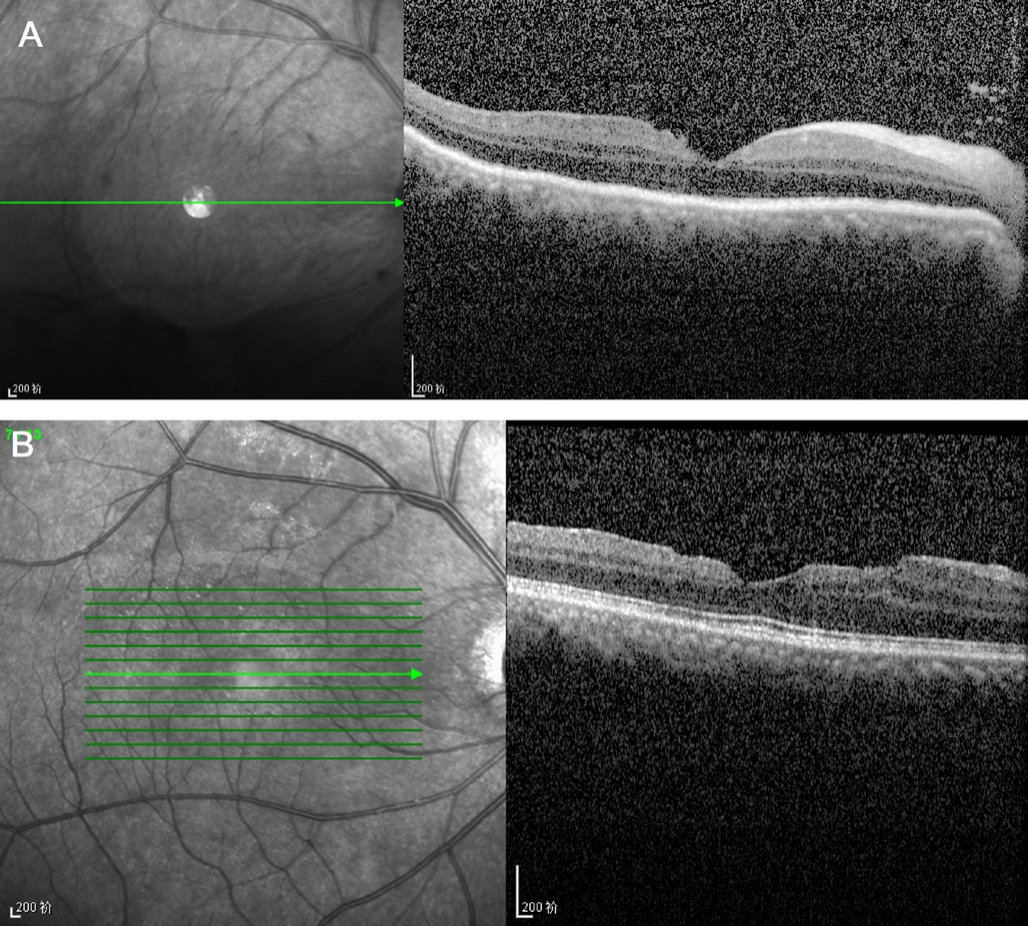
Postoperative orbital optical coherence tomography at 1 week (**A**) and 6 months (**B**), demonstrating preserved macular architecture with no separation of the retinal pigment epithelium or neurosensory retina.

**Figure 6:**
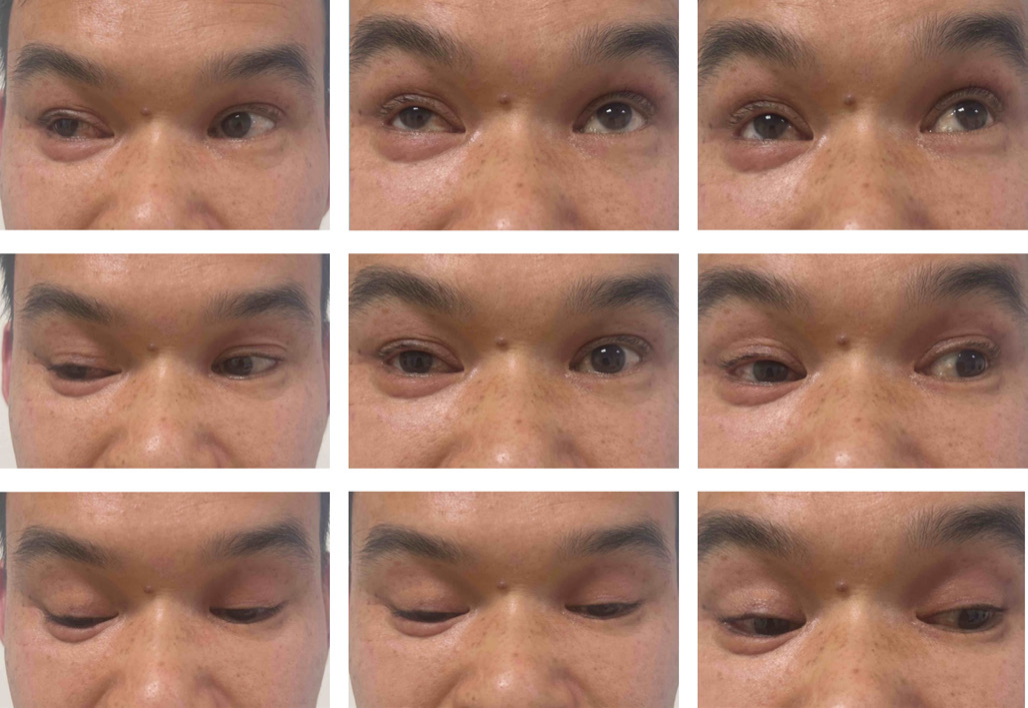
One-month postoperative nine-gaze motility chart showing largely recovered ocular motility, with only mild limitation of adduction.

Postoperatively, systemic anti-inflammatory and anti-infective treatments were administered. One week later, his right eye’s corrected visual acuity improved marginally to 20/200, Goldman visual field testing was normal and blood lead levels were within normal ranges. Ultra-widefield fundus photography (figure 4), optical coherence tomography (figure 5A,B) and B-scan ultrasound revealed vitreous haemorrhage and retinal detachment, with the macula attached, for which vitrectomy and silicone oil injection were performed. A small scleral laceration discovered intraoperatively was repaired with interrupted 8–0 nylon sutures. One month later, ocular motility had largely recovered; nine-gaze testing revealed only mild adduction limitation (figure 6). Three months later, the silicone oil was removed, and his corrected visual acuity improved to 20/100.

## Discussion

Orbital foreign bodies pose significant risks, including intracranial penetration, brain injury and infection.^3 4^ Imaging selection should be based on the nature of the foreign body—metallic objects often cause CT artefacts, making X-rays a valuable diagnostic alternative.^5 6^ The surgical approach should be carefully planned, avoiding blind extraction attempts, as forcibly removing an embedded object may exacerbate vascular or neurological damage. Instead, the endoscopic navigation-guided approach enables controlled exposure, precise localisation and removal of the large foreign body with minimal orbital trauma, ensuring safer outcomes. Additionally, even when the globe remains structurally intact, intraocular complications such as retinal detachment should not be overlooked, necessitating thorough postoperative monitoring.^7^ Peripheral retinal detachment and vitreous haemorrhage are considered the main contributors to the patient’s residual decreased vision despite prompt surgical intervention in this case. Finally, public education on eye protection, particularly for fishing enthusiasts, is crucial to prevent similar injuries.

**Learning points**

- Foreign bodies should not be forcibly extracted. A thorough examination is required first, and if initial removal attempts fail, an endoscopic navigation system should be used for careful exposure and dissection.
- Even if the globe remains intact, intraocular injuries such as retinal detachment should not be overlooked, highlighting the need for thorough postoperative ophthalmic evaluation.
- Postoperative blood testing for toxic element exposure, such as lead levels in this case, is essential to prevent systemic toxicity from embedded foreign bodies.
